# Health Disparities in Advance Care Planning: Development of a Spanish-Language LEAD Guide (Life-Planning in Early Alzheimer's and Other Dementias)

**DOI:** 10.1089/heq.2022.0143

**Published:** 2023-08-25

**Authors:** Kara Dassel, Rebecca L. Utz, Ana Sanchez-Birkhead, Sara Carbajal-Salisbury, Jeannette Villalta, Moroni Cajavilca, Lauren Solkowski, Nancy Aruscavage, Kathie Supiano, Eli Iacob

**Affiliations:** ^1^ College of Nursing, University of Utah, Salt Lake City, Utah, USA.; ^2^ Department of Sociology, College of Social and Behavioral Sciences, University of Utah, Salt Lake City, Utah, USA.; ^3^ Alliance Community Services, Murray, Utah, USA.

**Keywords:** Spanish, older adults, health disparities, goals of care

## Abstract

**Introduction:**

The LEAD (Life-Planning in Early Alzheimer's and Other Dementias) Guide is an advance care planning (ACP) tool for use within the context of dementia. To meet the needs of diverse communities, we sought to create a culturally sensitive and translated Latin American Spanish version of the guide.

**Methods:**

First, the guide was translated into Spanish. Second, we conducted forward and backward translations. Third, focus groups with Spanish-speaking Latino adults were held (healthy adults and current or previous dementia caregivers).

**Results:**

Descriptive analysis revealed three domains regarding the Latin American Spanish version of the LEAD Guide (LA LEAD Guide): (1) Family Dynamics (e.g., preventing family conflict), (2) Cultural Expectations (e.g., familial caregiving responsibility), and (3) Health Literacy (e.g., lack of knowledge about ACP).

**Discussion:**

This process created the Latin American LEAD Guide as a culturally and linguistically appropriate and acceptable ACP tool for older Latino adults.

**Health Equity Implications:**

The availability of culturally sensitive and Spanish ACP resources could facilitate greater health care access and research participation among Latino Americans by diminishing the linguistic and health literacy barriers for those not comfortably proficient in English.

## Introduction

Preparation for the end-of-life is a significant area of interest for clinicians and researchers, especially as our population ages.^1,2^ Advance care planning (ACP) is a communication process that empowers adults of any age and state of health to articulate and share their values, life goals, and preferences regarding future medical care.^3^ ACP is essential for persons with Alzheimer's disease or related dementia (ADRD), as end-of-life health care decisions often rely on the substituted judgment of family care partners^4^ after the individual with dementia loses decision-making abilities.^5,6^

Research has shown that ACP is most effective when one completes formal documents after conversing with future surrogate decision-makers.^7^ However, estimates show that only half of all adults engage in comprehensive ACP,^8,9^ and for Latinos, <20% participate in ACP activities.^10^ Other groups with low participation rates are those who were never married, of diverse racial and ethnic backgrounds, have low socioeconomic status, and have an ADRD diagnosis.^9,11,12^

To address the need for dementia-focused ACP, our team developed a tool called the LEAD Guide (LEAD stands for Life-Planning in Early Alzheimer's and Other Dementias).^13–15^ The LEAD Guide is value based rather than focused only on documenting specific medical decisions,^16–18^ and anticipates the need for surrogate decision-makers upon losing decisional abilities in the individual with ADRD.^17,19,20^ The LEAD Guide is the first dementia-focused ACP tool that used established instrument-development procedures to determine psychometric validity and reliability.^13^ However, the LEAD Guide was previously only available in English, which does not meet the needs of our growing diverse population, which will comprise 20% of the older adult (age 65+) U.S. population^21^ and experience a sixfold increase in ADRD by 2050.^22^ Developing culturally and linguistically appropriate health care resources and information is essential to prepare for the growth in demographic diversity and ADRD prevalence rates.

Therefore, the current study aimed to build upon existing ACP models targeted toward the Latino/Hispanic population by developing a culturally sensitive Latin American Spanish version of the LEAD Guide (LA LEAD Guide) by addressing common barriers to ACP, such as limited health literacy and differing cultural views on family involvement in care choices.^10^ For this study, we define Latin American Spanish as the language spoken primarily in Mexico and Central and South America. We used Latino and Hispanic interchangeably in this study because community stakeholders (community health and outreach workers) and participants felt comfortable with these terms.

Drawing upon the Ecologic Validity Model, we incorporated the eight dimensions to adapt the LEAD Guide to our study population culturally.^23^ The Ecological Validity Model developed by Bernal et al. for the cultural adaptation of psychosocial interventions recommends using eight dimensions to guide adaptations across language, persons, metaphors, content, concepts, goals, methods, and context. Adapting tools across these eight dimensions suggests increased cultural appropriateness and fit for Hispanic/Latino populations.^24^ The concepts of language, persons, context, and metaphors were key in our study as we understood that the Hispanic/Latino population consists of many subgroups. Hispanic subgroups have their unique cultural expressions and nuances in local language usage.

We were interested in adapting the LEAD Guide to be acceptable, culturally, and linguistically appropriate for Mexico and Central and South American peoples.^25^ Our collaboration with Latina researchers, community leaders, and health care workers with vast experience with Utah's Hispanic/Latino population guided the dimensions of content, concepts, goals, and methods. These dimensions refer to the content of cultural knowledge, beliefs, and values shared by ethnically diverse groups. Working closely with members and leaders from the Hispanic/Latino community allowed us to ensure that the LEAD Guide represented the culture-specific values of this population. Lastly, our collaboration also afforded us the confidence that congruency and consideration were given to developing culturally tailored tools and procedures customized for our participants' diverse and rich cultures.

## Materials and Methods

### Study design

This study (1) translated the LEAD Guide into a Latin American Spanish dialect using a multistep translation process and (2) used focus groups to assess the applicability and acceptability of the Spanish language version of the LEAD Guide. All procedures were reviewed and approved by the University of Utah Institutional Review Board (IRB_00136221). The study was deemed exempt from obtaining written informed consent. However, we provided participants with a consent cover letter. They consented verbally both at the time of recruitment and before the start of the focus group by a community health worker (J.V.).

### Translation process

**FIG. 1.**
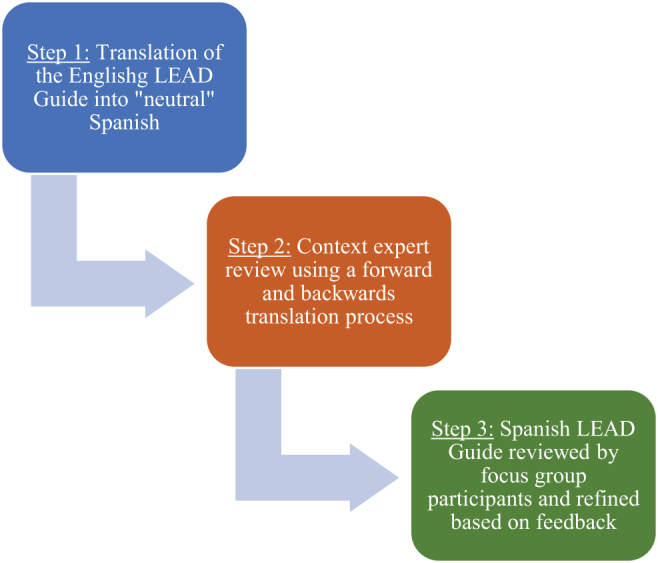
LEAD Guide translation process. LEAD, Life-Planning in Early Alzheimer's and Other Dementias.

The Cultural Adaptation Framework supported our methodological approach.^26^ We used a multistep approach to translation,^27^ where we conducted a *forward translation* of the questionnaire from its original language (English) to its target language (Spanish), followed by a *back-translation* from the target language to the original language.^28^ This forward–backward translation process focused on identifying ambiguous wording, concepts, and idiomatic expression, harmonizing across different Latin American subgroups' languages.^29^ The linguistic translation goal,^30^ while cross-cultural adaptation (e.g., ongoing collaboration with key stakeholders) and validation, were also considered^26,31^ (Fig. 1).

In the first step, we began with a forward translation of the English version of the LEAD Guide into “neutral” Spanish (meaning Spanish that can be understood by anyone that speaks Spanish regardless of country of origin by removing any country-specific Spanish words, idioms, etc.). A bilingual Latina community outreach worker (country of origin Guatemala) specializing in dementia care conducted this first translation. This process aimed to create a verbatim translation with a word-for-word similarity between the original and translated versions.

The second step consisted of a content-expert review by two Latino health care professionals (country of origin Mexico) and one community outreach worker (country of origin Guatemala), who systematically reviewed the translation using a forward–backward translation (a process where they compared the meaning and cultural relevance of the translated guide to the original English version). Each reviewer noted any inconsistencies, ambiguities, and errors in the translation, and then, based on the consensus of the review team, we made further revisions. This process aimed to iteratively create a more effective translation where one measures similar constructs across two languages.^32^

The third step assessed the applicability and potential use of the translated LEAD Guide by engaging the aid of a diverse group of Spanish-speaking Latino Americans. We used focus groups to facilitate this process. We asked participants about semantics, word choice, and a general understanding of the translated guide. The conversations noted difficulties in comprehension and identified areas where there was a need for further reconciliation in the translated materials.^33,34^ Focus groups were conducted, virtually, in Spanish and lasted ∼1.5 h each.

### Participants

The 14 focus group participants, on average 60.5 years old, mostly female (93%), well educated, spoke Spanish, were mostly immigrants and self-identified as Hispanic American or Latino (e.g., born in Argentina, Colombia, Peru). See Table 1 for details. We separated participants into two groups: (1) those who were current or former caregivers to persons with dementia (*N*=7), and (2) those who did not have first-hand experience with cognitive impairment or dementia caregiving within their families (*N*=7). Our team recruited participants through in-person outreach, including going out into the community, discussing the study, and assessing the eligibility of potential participants. Participants were compensated with a $100 gift card for participating in the focus groups. Participants in both groups were asked the same set of questions regarding the Spanish LEAD Guide (Table 2).

**Table 1.** Demographic Information on Focus Group Participants

Group	Age	Sex	Education	Country of origin	Years in the United States
Community participants					
Participant 1	82	Male	College degree	Peru	3
Participant 2	56	Female	High school degree	Mexico	35
Participant 3	67	Female	College degree	Argentina	39
Participant 4	51	Female	High school degree	Mexico	18
Participant 5	65	Female	College degree	Argentina	43
Participant 6	53	Female	High school degree	Mexico	1
Participant 7	61	Female	College degree	Chile	22
Dementia caregivers					
Participant 8	39	Female	High school degree	Mexico	6
Participant 9	48	Female	High school degree	Mexico	12
Participant 10	74	Female	College degree	Peru	1
Participant 11	69	Female	Some graduate school	Peru	45
Participant 12	76	Female	Some college	Peru	5
Participant 13	61	Female	College degree	American Mexican	61
Participant 14	45	Female	College degree	Argentina	10

**Table 2.** Focus Group Questions

Questions
(1) How long did it take you to fill out the document?
(2) Were there any questions that were not clear to you?
(3) Are there words you didn't understand?
(4) Were there any questions that you felt did not apply to you?
(5) Were there any culturally inappropriate questions?
(6) How can you foresee yourself using this document to help inform your care?
(7) Would you want to share this document with your doctor?
(8) Did filling out this document bring up any issues you had not previously considered? If so, which issues?
(9) Do you think completing this document will help you to have a conversation with your loved one about these topics? Why or why not?

### Analysis

We captured all focus group conversations verbatim, as uttered in Spanish. For qualitative analysis and data presentation, transcripts were translated into English by the bilingual community health worker facilitating the focus groups. A subset of the research team initially read through the transcripts for purposes of open coding. This coding resulted in the inductive identification of three codes that captured the topics addressed during the focus group conversations.

Next, the research team independently conducted confirmatory (deductive) coding of the transcripts. Each member read the transcripts line-by-line to identify any excerpts (i.e., set of comments that hang together into a single idea^35^) that fit into one of the three original open codes. Only quotes that received independent endorsement from at least two reviewers were confirmed. During this second stage of confirmatory coding, a fourth code emerged (i.e., specific revision suggestions).

## Results

We define the four themes identified from this two-stage hybrid approach to descriptive qualitative analysis in Table 3, with the most exemplary of the (translated) excerpts reported below. These four themes guided the final translation and refinement of the Spanish Latin American LEAD Guide. We found no discernible trends or differences among the caregiver versus the noncaregiver focus groups regarding thematic content or frequency of utterances; however, below, we have tried to identify whether an excerpt was from a caregiver or noncaregiver participant.

**Table 3.** Four Themes Describing the Use of the Life-Planning in Early Alzheimer's and Other Dementias Guide in a Spanish-Speaking Latino Population

Specific suggestions	Specific ways the Guide could be improved through language/translation, editing, and formatting. 44 Total excerpts: 22 from caregiver group, 22 from noncaregiver group)
Support for family	Using the Guide could help prevent family conflict, designate a health care proxy, and reduce burden of families facing ADRD. (39 Total excerpts: 19 from caregiver group, 20 from noncaregiver group)
Health literacy	A general lack of knowledge about advance care planning conversations, documentation, and dissemination. (38 Total excerpts: 20 from caregiver group, 18 from noncaregiver group)
Cultural nuances	Acknowledgment of cultural nuances between Spanish-speaking countries, the familial responsibility of caring for family in the home, and the influence of religion on end-of-life care decisions. (25 Total excerpts: 11 from caregiver group, 14 from noncaregiver group)

ADRD, Alzheimer's disease or related dementia.

### Specific revision suggestions

Findings from the focus groups identified specific areas for improvement. These suggestions focused on additional translation changes to meet the needs of the various Latin American regions. Some of these changes included exact wording or awkward translation.

For example, across both focus groups, there were conversations about cognitive impairment and dementia in the Spanish language. Ultimately, we chose the terms “perdida de memoria” (memory loss) and “demencia” (dementia) because they had the most significant universal recognition in the Spanish language and Latin American cultures. However, such words were sometimes (mis)interpreted or imbued with cultural connotations that were not intended (e.g., crazy, insanity, madness). Similarly, some participants insisted we change or delete questions that used terminology associated with nursing homes (interpreted as “asylums”), which are not viable care options for Latin Americans. Both caregiver and noncaregiver participants suggested terminology related to “rest homes” or “places of retirement.” Thus, we expanded the terms to describe a broader array of residential long-term care options since discussing preferred and nonpreferred locations for care and place of death in ACP is critical.

Across the two focus groups, there were similar suggestions to (1) add new topics related to life insurance, finances, and estate planning, (2) allow multiple family members to participate in the process of completing the guide (as opposed to a single, primary care partner), and (3) the identification of specific areas where questions/topics seemed redundant.

Participants found the document's Spanish language version valuable and easy to use. For example, one participant from the caregiver group recognized the quality of the paper translation and suggested that it had achieved a universal form of the Spanish language, “We are from different countries, and we could all read it. That is, the document is readable.” Participants from the other focus group agreed that the LEAD Guide was “quite understandable and quick to read” and that “this is pretty clear, pretty clear, and useful.” These positive overall assessments suggested that our systematic process to achieve an effective Spanish translation was successful.

### Support for family

Among the most commonly expressed reasons participants felt the document was helpful was that due to the opportunities it provided for families to engage in ACP conversations. One noncaregiver said,

It's good, uh, to do it, I mean, to read it, but also share it with those that surround you. Because right now, you're conscious. But when you're no longer aware, when other people have to make your decisions for you … It is good we make things clear now, what we want, what we decide, for when we can't think alone.

Another participant caring for a mother with “dementia” commented on how hard it was for her family to care for their mother because they did not initially think about asking her about her needs and wishes. Now it is too late to ask her. She mentioned that the LEAD Guide would be helpful if completed with a “family approach.” Another caregiver concurred,

I found the guide very useful, because personally, I think it takes the responsibility, and even the blames from relatives if they have to make a decision. Because we are making decisions about ourselves, I think it is a duty, and it is a responsibility. Now, if we help other people, we also take off a weight, because it is the person who expresses his desires, and that also gives us peace of mind.

A woman in the caregiver focus group emphasized the importance of having resources and educational materials available in all languages: “ah, when my parents died. I was translating it, and it took us hours. It would have been marvelous to have this beforehand.” By drawing on the strong sense of familism within Latin American cultures, other participants reinforced the importance of a document such as this: “The impact [of a resource like this] is very big, because it really is something we need to think about and help. Helping our family is something very important.”

### Health literacy

Regardless of whether they had experience with dementia caregiving, focus group participants were not all that familiar with ACP. One noncaregiver commented, “I've never read anything like that.” A caregiver said, “I'll tell you, for me, the truth is I am just hearing about these kinds of documents.” Similarly, some were confused or questioned about the difference between general ACP conversations and more formal legal documentation such as advance directives and wills. Others were unsure if they were required to share ACP with their health care provider (whom they may or may not trust) or whether sharing the information would impact their future care. These conversations often circled back to the cultural belief and value that Latino families should be the care partners for those needing help.

This belief aligns with past research that found that regardless of race/ethnicity, families are often unaware of ACP processes, concepts, or legal documents,^8,9^ but typically assume the responsibility to provide care to others, even in strained relationships.^36,37^

Perhaps unique to Latin American cultures, especially among those who have immigrated to the United States within their lifetimes, there was a focus on documenting and legalizing these types of ACP conversations among participants of both focus groups. For example, we received several suggestions to modify the LEAD Guide so that it could become a legal document: “change it into a notarized letter” and “you just need to designate a relative or someone who you think is qualified for it and you are ready—get it notarized, and ready for use.”

The LEAD Guide is intended to facilitate conversations about goals of care but is not a legal document. However, in response to these conversations, we edited the LEAD Guide's introductory materials and instructions to reinforce the difference between ACP conversations (such as those facilitated by the LEAD Guide) and formal documents that would define legal issues related to power-of-attorney and inheritance, for example.

The LEAD Guide includes a glossary to address the expected low literacy levels around ACP topics. One of the noncaregiver participants reflected that although medical terminology was not something she was familiar with, she felt the document did a nice job explaining those terms in a way that made it more accessible, “Things could be simplified a little more for those who do not have a medical vocabulary—although the glossary was divine, really.” Another respondent concurred that the document, especially because of its clear and universal translation, was accessible to everyone: “To me, there is an aspect that is transcendental. I think the questions are asked for all kinds of people. Those with different ideologies, with, in, different religions, and economic life. … It is like for all different kinds of people.”

In recognition of their overall lack of awareness about ACP topics and a positive assessment of the LEAD Guide, participants in both focus groups expressed a strong need for a resource such as this: “it's something we need to educate ourselves and educate our community about,” and

It should be filled out among all people, I hope that you, [Name of the community service organization that facilitated the focus groups], will do a training for families so that they will know the existence of this document. It would be great if the entire population could access that. That is, the whole Hispanic community could access this kind of documentation more easily because it is informative and very good, very clear.

These comments reflect a lack of access and awareness of ACP resources among Latin American populations, as reported in previous research.^38,39^

### Cultural nuances

One participant commented, “As you target the Hispanic population, you always have to contemplate that the psychology of Hispanics is different from that of people here,” which prompted us to ask specifically how the psychology and mentality were different. Across both focus groups, participants identified a strong sense of familism in which a higher emphasis is placed on the family unit in terms of respect, support, and obligation^40^: “We Latinos are like very closely knit, very sensitive, very family oriented” and “We Hispanics usually take care of our relatives. It is very natural for us. … And we do … as the heart dictates to us.” In caregiver and noncaregiver groups, others described traditional gender-based expectations that assumed that Latino men were more likely to make the decisions.

In contrast, Latino women are the ones that typically provide the care. And still, others focused on the larger family sizes among Latinos (compared with white families) and the greater tendency for Latino families to coreside with 3+ generations.

## Discussion

This article describes the systematic process of developing and adapting the LA LEAD Guide, an ACP tool used to guide conversations about an individual's care values and preferences across the dementia trajectory.^13^

Participation in ACP conversations and documentation is essential in providing end-of-life care that aligns with patient wishes^7^; however, ACP is grossly underutilized by persons from a diverse racial and ethnic background (e.g., Latinos), with low socioeconomic status, or with a dementia diagnosis.^9,11,12^ Similarly, Latinos are not as likely to participate in research studies, which has led to a poor understanding of ADRD in the Latino population relative to the non-Latino white population.^41^

Many of the specific comments we received through our translation and refinement process reflected potential cultural differences unique to Spanish-speaking Latino Americans. These were addressed through our systematic translation and refinement process, creating a culturally relevant translation of the LEAD Guide. The research team *affirmed* the final selection of (terminology/terms/questions/order of questions/instructions) through incremental steps, including review by content experts, review by language experts in various forms of Spanish usage, and incorporation of recommendations from focus group participants. The research team adjudicated each modification to achieve consensus on the present version. For example, the LEAD Guide addresses shared decision-making among Latinos identified in the theme of *Support for Family*. We added information in the instructions to include as many family members as preferred in ACP conversations facilitated by the LEAD Guide.

Thus, the methodical and thoughtful translation process, including the analysis of focus group data, allowed us to create a more effective and accessible version of the LEAD Guide that is both culturally specific and relevant and reflects diverse family experiences and values.

The themes observed in this study are mostly unique to those that emerged in developing the English LEAD Guide.^13^ Analysis of focus group feedback in the current study (which included community members and caregivers of persons with dementia) was culturally specific. These themes included specific linguistically appropriate language revisions, the need for greater health literacy surrounding ACP, addressing family support (familism), and gender-based cultural nuances. Our previous work's analysis of focus group feedback (which included caregivers of persons with dementia and individuals with cognitive impairment) focused on the instructions, wording, and formatting of the guide rather than cultural domains. In both groups, participants were enthusiastic about the guide and the need for such a tool to help families engage in ACP conversations, particularly in dementia.^42^

Full-text versions of both the English and Spanish versions of the LEAD Guide are free and publicly available online (http://utahgwep.org). Next, our team is designing and implementing national intervention studies, utilizing both versions of the LEAD Guide to provide information, support, and instructions to guide diverse families through the ACP process to create an informed transfer of knowledge from the care recipient to the care partner. The careful processes used to translate and culturally adapt the ACP intervention tools (as described herein) are just one of the steps our team is using to integrate health equity into developing, refining, and evaluating evidence-based intervention materials that can be delivered and implemented in a dementia-focused population.

We recommend that researchers consult the best practices and resources developed by the National Institute on Aging's IMPACT Collaboratory to ensure that health equity is a guiding principle of the research efforts used to inform dementia care practices.^43^

This study has limitations. First, we held focus groups using videoconferencing software. This modality may have resulted in a selection bias toward participants who have access to a computer and the internet and know how to utilize the associated technology and software. Second, our community partners recruited, ran, and translated the focus group sessions, which may also introduce bias into the study design. Third, we had a limited number of well-educated focus group participants, and so, the findings are not generalizable to the vast heterogeneity of the Latin American population. Future studies include the need to conduct additional focus groups with more heterogeneous participants, including persons who have experience with ACP.

## Health Equity Implications

The availability of culturally sensitive and Spanish ACP resources could facilitate greater health care access and research participation among Latino Americans by diminishing the linguistic and health literacy barriers for those who are not proficient in English. With a rapidly growing and aging Latino population and the low levels of ACP completed within diverse communities, the LA LEAD Guide has the potential to fill an essential and significant health care gap.^21,41,44^ The LEAD Guide was created to guide families through ACP conversations, hopefully reducing the burden felt by family members called upon to be proxy decision-makers for family members with dementia.

## Abbreviations Used

ACP: advance care planning
ADRD: Alzheimer's disease or related dementia
LA LEAD Guide: Latin American Spanish version of the LEAD Guide
LEAD: Life-Planning in Early Alzheimer's and Other Dementias
